# Formation and Analysis of Volatile and Odor Compounds in Meat—A Review

**DOI:** 10.3390/molecules27196703

**Published:** 2022-10-08

**Authors:** Julian Bleicher, Elmar E. Ebner, Kathrine H. Bak

**Affiliations:** 1 Institute of Food Safety, Food Technology and Veterinary Public Health, University of Veterinary Medicine Vienna, Veterinärplatz 1, 1210 Vienna, Austria; 2 FFoQSI GmbH—Austrian Competence Centre for Feed and Food Quality, Safety and Innovation, Technopark 1C, 3430 Tulln, Austria

**Keywords:** aroma, meat, odor, dynamic headspace, GC-O, GC-MS, SBSE, SPME, electronic nose, sensorial analysis

## Abstract

The volatile composition and odor of meat and meat products is based on the precursors present in the raw meat. These are influenced by various pre-slaughter factors (species, breed, sex, age, feed, muscle type). Furthermore, post-mortem conditions (chiller aging, cooking conditions, curing, fermentation, etc.) determine the development of meat volatile organic compounds (VOCs). In this review, the main reactions leading to the development of meat VOCs such as the Maillard reaction; Strecker degradation; lipid oxidation; and thiamine, carbohydrate, and nucleotide degradation are described. The important pre-slaughter factors and post-mortem conditions influencing meat VOCs are discussed. Finally, the pros, cons, and future perspectives of the most commonly used sample preparation techniques (solid-phase microextraction, stir bar sorptive extraction, dynamic headspace extraction) and analytical methods (gas chromatography mass spectrometry and olfactometry, as well as electronic noses) for the analysis of meat VOCs are discussed, and the continued importance of sensorial analysis is pinpointed.

## 1. Introduction

Flavor is, obviously, of vital importance for the eating quality of meat. It has been suggested that flavor be defined as a term used to describe the combined influences of taste, odor, the trigeminal system, and touch, with the addition of visual and auditory cues [1]. This is well in line with the ISO definition of flavor, which states that flavor is a “complex combination of the olfactory, gustatory and trigeminal sensations perceived during tasting” [2].

Whereas taste refers to the five basic tastes—sweet, sour, salty, bitter, and umami—odor refers to the aroma elicited by certain volatile compounds [3]. It is well known that raw meat has a bland flavor with little aroma. However, raw meat contains numerous precursors of meat flavor, which result in the formation of volatile odor compounds, especially during cooking [3,4]. Several hundred or even thousand volatile compounds have been identified in meat from various species [3,4,5]. The odor of meat from different species has many volatile compounds in common [6], though often with quantitative differences between species [4] as well as species-specific odor compounds [7].

The reactions leading to the formation of meat aroma include, for instance, lipid oxidation, Maillard reaction, Strecker degradation, thiamine degradation, and carbohydrate degradation as well the interactions between reaction products, [3] resulting in volatile compounds representative of most classes of organic compounds: hydrocarbons, alcohols, aldehydes, ketones, carboxylic acids, esters, including lactones, ethers, furans, pyridines, pyrazines, pyrroles, oxazoles and oxazolines, thiazoles and thiazolines, thiophenes, and other sulfur- and halogen-containing compounds [8] (as cited by [4]).

When evaluating meat odor, both the qualitative and the quantitative properties must be considered, in addition to the possible synergy between different aroma compounds [4]. It must also be kept in mind that simply because a volatile compound is present in a high concentration does not necessarily mean that it is significant for the odor. The so-called odor activity value (OAV) determines the contribution of a volatile compound to the odor of the food, and is defined as the ratio of the concentration of the volatile compound in the food divided by the odor threshold of the compound in an appropriate matrix (water, air, or even a more complex matrix [9,10]).

The composition of the food matrix (moisture content, pH, presence of fat and protein, etc.) needs to be given serious consideration when deciding on an appropriate method for analysis, as do the volatile compounds of interest and their potential ranges in the sample [11].

In this review, the formation of volatile and odor compounds in meat and meat products will be described, followed by a discussion of pre-slaughter and post-mortem factors affecting meat (product) odor. Finally, the most common methods for the determination of volatile compounds in meat will be described and their usefulness and limitations will be discussed.

## 2. Odor Compounds in Meat

### 2.1. Formation of Odor Compounds

The cooking of meat results in the formation of volatile compounds from water-soluble precursors as well as lipids [5]. The main water-soluble precursors are amino acids, peptides, glycogen, reducing sugars, nucleotides, and thiamine [3,4,5]. The most important reaction pathways are described below, and the main classes of volatile compounds formed during the cooking of meat are shown in Table 1.

#### 2.1.1. Maillard Reaction and Strecker Degradation

The Maillard reaction, also known as non-enzymatic browning, is a reaction taking place between a free amino group of an amino acid and the carbonyl group of a reducing sugar [12].

**Figure 1:**
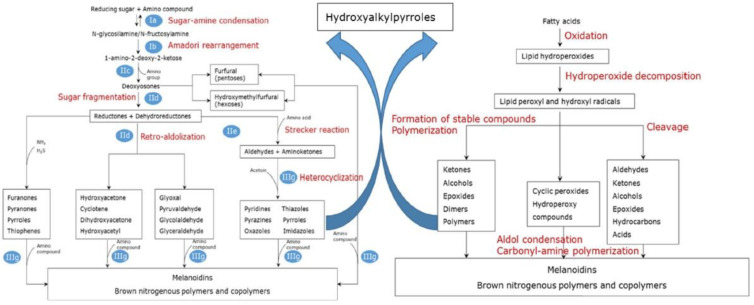
Overview over the routes of formation and common volatile compounds formed by the Maillard reaction and Strecker degradation, lipid oxidation, and their interaction. Reprinted from Diez-Simon et al. (2019) [14] with permission from Springer Nature under the terms of the CC BY license.

The initial stage of the Maillard reaction is the condensation between the amino group and the reducing sugar to form a glycosylamine, followed by a rearrangement to form either an Amadori compound (if the reducing sugar is an aldose) or a Heyns compound (if the reducing sugar is a ketose) [5,12,13]. The Amadori or Heyns rearrangement products are then dehydrated and degraded via deoxyosones in the intermediate stage [5,13], generating furanones, furfurals, and dicarbonyl compounds [3,5]. In the final stage, these compounds react with other reactive compounds such as amines, amino acids, aldehydes, hydrogen sulfide, and ammonia, resulting in the formation of meat flavor compounds belonging to various classes [5] (Figure 1).

Related to the Maillard reaction is the Strecker degradation, which may be considered a subset of the Maillard reaction, and is a crucial step for meat flavor generation [15]. Strecker aldehydes, which are odor compounds in cooked meat, are formed via the reaction between an amino acid and an α-dicarbonyl compound (e.g., a deoxysone [13]). This reaction generally forms a Strecker aldehyde (which has one less carbon atom than the original amino acid), carbon dioxide, and an α-aminoketone [15,16]. The α-aminoketones serve as important flavor precursors, leading to the formation of various classes of heterocyclic compounds important for meat flavor [16].

Overall, the Maillard reaction and Strecker degradation result in the formation of N-, S-, and O-heterocyclic compounds as well as other sulfur-containing compounds, all of which are important meat flavor compounds [16]. The compounds include furans, furanones, methylfuranthiols, imidazoles, pyrazines, pyridines, pyrroles, oxazoles, thiazoles, thiophenes, alkanethiol, alkyl sulfides, and disulfides (see Table 1 and Figure 1).

**Table 1:** The main classes of volatile compounds formed during the cooking of meat.

Volatile Compound Class	Chemical Structure	Formation	General Odor Descriptors [17] ^1^
Alcohol	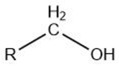	Lipid oxidation [5] Carbohydrate fermentation ^2^ [17]	Saturated alcohols: high threshold. Straight-chain primary alcohols: flavorless. Increase in carbon chain: stronger flavor—greenish, woody, fatty floral. Unsaturated alcohols: mushroom, green leaf, musty.
Aldehyde	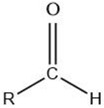	Lipid oxidation [5]	C3 and C4: sharp and irritating. C5–C9: green, oily, fatty, tallow. C10–C12: citrus orange peel. Alkyl-branched aldehydes: malty.
Carboxylic acid	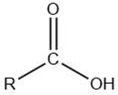	Lipid oxidation [5]	Saturated acids: acidic. Unsaturated branched-chain acids: pungent, sour, penetrating. Keto acids: burnt, caramel, sour.
Ester	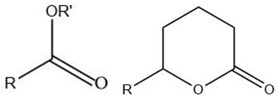	Lipid oxidation [5]	Esters from C1–C10 acids: fruity sweet. Esters from long-chain fatty acids: fatty flavor.
Ketone	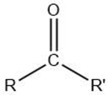	Lipid oxidation [5]	Unsaturated ketones: animal fat odors. 2-Alkanones: spicy, fruity, fatty, citrus-like. Lactones: oily, fruity, buttery, fatty.
Furan	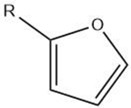	Maillard reaction [16] Lipid oxidation [5] Carbohydrate degradation [14]	Alkylfurans: grassy, beany odor.
Furanone	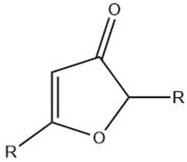	Maillard reaction [5]	Fruity, fatty, roasty, roasted almonds, sweet aroma, pungent [11].
Imidazole	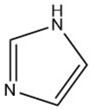	Maillard reaction [16]	Amine-like [pubchem.org].
Methylfuranthiol	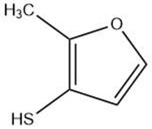	Maillard reaction [5] Thiamine degradation [15]	Meaty aroma, roast meat, boiled meat [5,15].
Oxazole	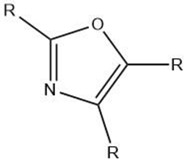	Maillard reaction [16] Interaction ^3^ [14]	Green and vegetable-like [16].
Pyrazine	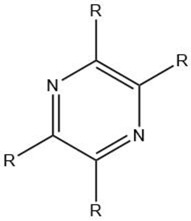	Maillard reaction [12] Interaction ^3^ [14]	Pleasant aroma: nutty roast aroma, earthy, potato-like.
Pyridine	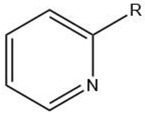	Maillard reaction [12] Interaction ^3^ [14]	Fatty tallow aroma.
Pyrrole	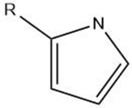	Maillard reaction [12]	Burnt earthy odor.
Thiazole	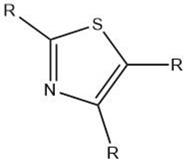	Maillard reaction [14] Interaction ^3^ [14]	Green, vegetable-like, nutty, roasted.
Thiophene	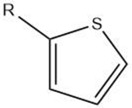	Maillard reaction [16] Interaction ^3^ [14] Thiamine degradation [12] Nucleotide degradation [14]	Meaty aroma.
Alkanethiol	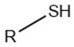	Maillard reaction [5] Thiamine degradation [14]	Meat-like, sulfurous, cabbage, onion, garlic-like. Associated with boiled meat.
Alkyl sulfide	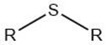	Maillard reaction [14] Thiamine degradation [14]	
Alkyl disulfide	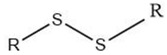	Maillard reaction [16] Thiamine degradation [14]	

^1^ Odor descriptors from [17], unless stated otherwise. ^2^ Microbial fermentation during the production of dry-cured meat products [17]. ^3^ Interaction signifies the interaction between Maillard reaction products and lipid-derived aldehydes.

The importance of nucleotides for meat flavor development is also via the Maillard reaction [3]. Ribonucleotides are degraded by enzymatic dephosphorylation post-slaughter, which produces ribose from inosine monophosphate and 5′-ribonucleotides [18]. Ribose then participates in the Maillard reaction and is able to form a number of sulfur-containing volatiles [19].

#### 2.1.2. Lipid Oxidation

Lipid oxidation takes place in three stages—initiation, propagation, and termination. It begins with the formation of lipid hydroperoxides, which are then decomposed into peroxyl and hydroxyl radicals. In the termination step, stable compounds are formed. These are then cleaved or polymerized into the final products [14]. Cooking promotes lipid oxidation [18], which results in the formation of several types of lipid oxidation and degradation products, including aliphatic hydrocarbons, aldehydes, ketones, alcohols, carboxylic acids, and esters, including lactones in addition to other aromatic compounds such as alkylfurans [5] (see Table 1 and Figure 1)—the specific products depending on the original fatty acid [14]. As phospholipids contain a significantly higher proportion of unsaturated fatty acids than triglycerides, it is not surprising that phospholipids are the most important lipid source of meat odor compounds [5], with species-specific odor compounds originating from the lipid fraction of the meat [7].

In general, the odor threshold values for the lipid oxidation-derived volatiles are much higher than those for the N- and S-containing compounds generated from the Maillard reaction, and their importance for cooked meat odor is, therefore, minor. Exceptions are the 6–10 carbon-saturated and -unsaturated aldehydes, which contribute important fatty flavors to cooked meat [5].

The interaction between lipid oxidation products and Maillard reaction products seems also to be important for the development of cooked meat flavor. It has long been speculated that lipid oxidation products inhibit the production of pyrazines [20]. It was later found that phospholipids and their degradation products inhibit the formation of certain heterocyclic compounds, importantly, certain S-heterocyclic compounds [16], which help balance the generation of cooked meat odor [16,21]. The aldehydes formed during lipid oxidation can take part in the Maillard reaction at both initial and later stages to form meat odor compounds. However, most of these lipid oxidation–Maillard reaction interaction-derived compounds have weak odor intensities and high odor thresholds, meaning that their importance for cooked meat odor is likely limited [16].

During the storage of meat and meat products, an unwanted degree of lipid oxidation known as warmed-over flavor (WOF) may occur and affect meat quality negatively [22,23]. WOF arises when previously cooked, refrigerated meat is reheated [24], and is characterized by off-odor and off-flavors described most commonly as “stale”, “cardboard”, “rancid”, and “metallic”, which have been related to hexanal, 2,3-octanedione, and total volatiles [25] as well as to trans-4,5-epoxy-(E)-2-decenal and to the loss of desirable furanones [24]. WOF has been ascribed to oxidation, mainly of phospholipids, during refrigerated storage and is catalyzed by both heme- and non-heme iron released during cooking [3,25,26].

#### 2.1.3. Carbohydrate Degradation

Upon heating, carbohydrates are degraded as part of the Maillard reaction as described above. However, they can also be broken down via microbial fermentation in fermented meat products to produce organic acids—most notably, lactic acid—as well as volatile compounds in the form of carboxylic acids and linear alcohols [17] (Table 1). Carbohydrate (sugar) degradation can also take place by caramelization, but as this only takes place at rather high temperatures, caramelization is only relevant for meat surface areas during grilling or roasting [3,12].

#### 2.1.4. Thiamine Degradation

**Figure 2:**
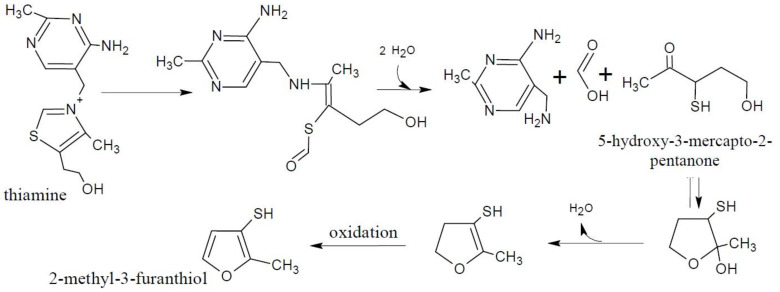
Thiamine degradation to the formation of 2-methyl-3-furanthiol via 5-hydroxy-3-mercapto-2-pentanone. Reprinted from Resconi et al. (2013) [15] with permission from MDPI under the terms of the CC BY license.

Thiamine is a sulfur- and nitrogen-containing vitamin [12,15]. Several studies using model systems have shown that thiamine is an important precursor of meat odor, with thermal degradation depending on temperature, time, pH, and the matrix composition [3]. The thermal degradation of thiamine into the final meat odor compounds is a complex reaction with various degradation pathways and numerous intermediate products before the production of the final meat odor compounds containing sulfur or nitrogen [12]. These include, for instance, alkanethiols, sulfides, and disulfides [14] (Table 1). An example of the thermal degradation of thiamine is shown in Figure 2.

In cooked ham, thiamine has been proven to play a key role in the formation of known meat odorants 2-methyl-3-furanthiol (Figure 2), 2-methyl-3-methyldithiofuran, and bis(2-methyl-3-furyl)disulfide [27]. The 2-methyl-3-methyldithiofuran from thiamine degradation is also a proven meaty off-odor compound in heat-treated fruit juices [28]. However, adding four times the physiological concentration of thiamine to raw beef resulted in no changes to the flavor of cooked beef, presumably due to a lack of phosphate [29], and similar results have been found for chicken [30]. Hence, some authors deduce that thiamine as a meat flavor precursor seems to be most essential for pork flavor [15].

Considering that many of the thiamine degradation products are still found as important meat aroma compounds in other species than pork, as well as in dry-fermented pork products (i.e., no heat treatment), it is not surprising that some of these odor compounds can likewise result from the Maillard reaction or Strecker degradation involving sulfur-containing free amino acids [15] or as a result of microbial metabolism [31].

### 2.2. Pre-Slaughter Factors Affecting Meat Volatile and Odor Development

Various pre-slaughter factors such as animal species, breed, sex, age, muscle type, and feed affect the generation of meat flavor precursors and, hence, the generation of volatile and odor compounds [3,32].

#### 2.2.1. Animal Species

The water-soluble precursors are responsible for the majority of the volatile organic compounds (VOCs) in cooked meat, as shown above. However, it is the lipid fraction which is responsible for species-specific flavor. Sensory panel studies carried out in 1968 confirmed previous studies on the volatile compounds of cooked meat from different species [33] (according to [7]). Due to their higher degree of unsaturation, the species-specific flavor has mainly been attributed to phospholipids more so than to triglycerides [5,34], which also explains the species differences in WOF development (in order of decreasing susceptibility: turkey, chicken, pork, beef, mutton) [35]. However, later research points in the direction that species-specific flavor is the result of a complex interaction between the lipid- and water-soluble compounds [5,21].

#### 2.2.2. Animal Breed

The animal breed has been shown to affect total fat content, intramuscular fat content as well as fatty acid composition [32]. For ruminants such as beef and sheep, species is a significant determinant of fatty acid composition, whereas for monogastric animals such as pigs, feed composition plays a much more important role [36]. An effect of breed on cooked meat flavor was found for beef, e.g. [37]. However, not all studies on ruminants found an effect of breed. For example, [38] found no effect of breed on the flavor of cooked lamb, and [39] only found a minor effect of breed on the flavor of grilled beef, but only two breeds were investigated in each study.

#### 2.2.3. Sex of the Animal

The effect of the sex of the animal on meat flavor precursors was reviewed by [32], who found that the amount of subcutaneous and intermuscular fat varies between sexes, with female animals generally having more fat. Differences in cooked meat flavor have been found for beef between heifers and bulls [37], whereas no difference was found for wethers and female lamb [40], nor for male and female foals [41].

The effect of castration on meat flavor is well known for pigs in the form of boar taint, which is mainly an unpleasant odor, but also an unpleasant flavor in meat from entire male pigs, caused primarily by androstenone (5a-androst-16-ene-3-one [42]; urine odor [43]) and skatole (3-methyl indole [42]; fecal/manure odor [3,43]), but also other volatile compounds [43,44]. Boar taint can be prevented through surgical castration or immunocastration (vaccination) [43]. Castration also affects the flavor of lamb, with the meat from rams having a more intense flavor than meat from wethers [38].

#### 2.2.4. Animal Age

As animal age increases, so does the intensity of the cooked meat flavor, as exemplified by the rather bland flavor of veal compared to beef [3] and the much milder flavor of lamb compared to mutton [45]. The flavor-intensity of beef has been found to increase up to 18 months of age, presumably due to age-related changes in the volatile compound precursors related to the increase in fat content and amount of saturated fat [3].

#### 2.2.5. Feed

The fatty acid composition of the meat can easily be manipulated through the feed for monogastric animals such as pigs, while doing the same thing with ruminants is more difficult due to the saturating effect of the rumen [46]. However, it is, nevertheless, possible to some degree [47].

The feeding of pigs and poultry to increase the amount of omega-3 fatty acids in the meat can be problematic due to the development of fishy off-odors and off-flavors, which need to be combatted by a higher content of antioxidants in the feed [46].

Meat from sheep contains species-specific short branched-chain fatty acids, particularly 4-methyloctanoic acid, 4-ethyloctanoic acid, and 4-methylnonanoic acid, which are responsible for the characteristic mutton aroma. The content of these short branched-chain fatty acids is influenced by the diet [32]. Feeding sheep grain-based concentrates as opposed to them being raised on pasture has been found to lead to an increase in the amount of short branched-chain fatty acids [48]. The effect of diet on the composition of aroma compounds in ruminant meat was reviewed by [49]. Additionally, here it was found that grain-based diets lead to a higher content of branched-chain fatty acids, in addition to certain aldehydes (e.g., hexanal [48] and other aldehydes originating from linoleic acid [49]) and lactones (δ-tetradecalactone and δ-hexadecalactone [50] according to [49]) as opposed to meat from grass-fed animals, which had higher contents of various phenols [48,49], terpenes [49], indoles [48,49], and sulfur compounds [49]. A higher content of terpenes, among other compounds, in grass-fed sheep was reported by [51]. Another study [39] found that feeding beef a cereal-based concentrate led to an increase in linoleic acid decomposition products among the volatile aroma compounds. On the other hand, feeding beef a grass silage-based diet resulted in a higher amount of α-linolenic acid decomposition products [39]. It has also been found that meat from grass-fed beef has a higher concentration of free amino acids than meat from concentrate-fed animals [52], which potentially leads to the generation of more pyrazines and Strecker aldehydes [3].

The accumulation of plant-derived compounds from the feed into the meat has the potential to influence the volatile composition of meat, as reported, e.g., for pork [43] and sheep [45]. It might be possible to determine the feeding background of an animal based on the analysis of the volatile compounds of the raw meat [53] as well as cooked meat, as reviewed by [32].

#### 2.2.6. Muscle Type

Flavor intensity as well as off-flavor intensity varies between muscles, as reviewed by [54]. Although differences in flavor intensity between muscles are evident due to biochemical differences [3], it should be mentioned that the differences in beef flavor between muscles are fairly minor [54].

### 2.3. Post-Mortem Factors Affecting Meat Volatile and Odor Development

Post-mortem factors affecting the development of meat flavor include the slaughter process as well as the subsequent carcass handling, chiller aging, cooking, and storage after cooking [55], as well as processing such as curing and fermentation [3] and preservative technologies such as irradiation [56].

#### 2.3.1. Chiller Aging

The slaughter process itself affects the early post-mortem biochemical events, including the pH decline and resultant ultimate pH [55], which is important due to the effect of pH on the Maillard reaction [54], including the increase in pyrazine formation at high pH and inhibition at low pH [57]. Additionally, pH affects the activity of the proteolytic enzymes (calpains and cathepsins) [58], with µ-calpain retaining 24–28% of its maximum activity (pH 7.5, 25 °C) when pH is lowered to 5.5–5.8 post-mortem [59].

During chiller aging, also known as conditioning or maturation, changes in tenderness but also meat flavor may occur [3]. The changes in meat flavor are, to a large degree, caused by increases in free amino acids and peptides [60,61]. Lipid oxidation [62] and nucleotide breakdown [60,63] also contribute to the flavor development during chiller aging. Nucleotide breakdown importantly includes the breakdown of inosine 5′-monophosphate to ribose [60], as mentioned earlier. As previously described, these flavor precursors take part in the Maillard reaction and Strecker degradation, and produce pyrazines and Strecker aldehydes [3].

Undesirable changes in the volatile composition during storage may be due to microbial spoilage. Certain alcohols, aldehydes, ketones, fatty acids, esters, and sulfur compounds are all potentially developed in meat during storage as a result of microbial spoilage [64]. The exact development of these undesirable volatile compounds depends on the storage conditions (temperature and packaging atmosphere, i.e., air, modified atmosphere, or vacuum) and on the strains of the microorganisms present [64]. Generally, anaerobic microorganisms produce off-odor compounds, which are more unpleasant than the odors produced by aerobic microorganisms [3]. The development of microbial off-odors during storage is illustrated by [64], where it is shown that, for instance, at the early stages of aerobic storage, fruity and dairy-like odors caused by esters and fatty acids develop, whereas these odors are not found during vacuum storage until much later stages of storage [64]. The main microorganisms responsible for microbial spoilage odor include *Pseudomonas fragi*, *Brochothrix* *thermosphacta*, *Enterobacteriaceae*, lactic acid bacteria (e.g., *Carnobacterium* spp., *Lactobacillus* spp., and *Leuconostoc* spp.), and *Clostridium* spp. [3,64]. For further details regarding which volatile compounds responsible for spoilage odor are produced by which bacteria under specific conditions, the reader is referred to [64].

#### 2.3.2. Curing

Curing is an old technology used for meat preservation, which also develops desirable cured flavor characteristics in addition to the characteristic cured color [65].

Very simply speaking, curing takes place by the addition of nitrite or nitrate to the meat, the latter then being converted to nitrite by nitrate reductase present in certain microorganisms [65,66]. Nitrite reacts with the meat pigment myoglobin, ultimately forming nitrosylmyoglobin, which is denatured to the stable nitrosylmyochromogen (stable except for in the presence of both light and oxygen [65]) due to either heat or low pH [66].

Cured meat products can be divided into cooked cured meat products and dry-cured meat products, which have some obvious differences in flavor profiles due to the different processing conditions [3]. It is well known that curing, to a large degree, prevents lipid oxidation [67,68]. The lack of lipid oxidation products is, at least partially, responsible for cured meat flavor [3]. This is in part due to the strong inhibition of formation of hexanal [3,68,69] and in part due to the general lack of lipid oxidation products, meaning no masking of meaty notes from sulfurous compounds [69].

Cooked, cured meat products, despite seeing a significant reduction in lipid oxidation compared to uncured, cooked meat, still obtain some of their flavor from lipid oxidation products. However, also other reactions related to heat treatment influence the flavor, these being amino acid breakdown, thiamine degradation, and Maillard reaction [69]. Key compounds that have been identified as suppressing the characteristic “cooked ham” odor of cooked, cured ham (as opposed to nitrite-free cooked ham) include 3-methylbutanoic acid (lactic-cheesy odor, origination from amino acid breakdown) and lipid oxidation products 1-octen-3-ol and 1-octen-3-one (mushroom odor), hexanal, octanal, nonanal, 2-nonenal, and 2-decenal (fruity-floral, vegetable, herbaceous, and/or chemical odors) [69].

Dry-cured meat products get their flavor from lipolysis (via lipases), enzymatic oxidation, and proteolysis (via proteolytic enzymes such as calpains and cathepsins) [70] as well as from lipid oxidation [17,18] and Maillard reaction due to the long ripening time and low water activity [17]. Key aroma compounds in dry-cured ham were reviewed by [17]. Important aldehydes include hexanal and 3-methylbutanal, while 1-penten-3-ol is an important alcohol. Other important compounds include the nitrogen compounds pyrrole, 2-acetyl-1-pyrroline, dimethylpyrazine, and tetramethylpyrazine, as well as the sulfur compounds dimethyldisulfide, methanethiol, and 2-methyl-3-furanthiol—the latter also known to be produced from the thermal degradation of thiamine [17].

#### 2.3.3. Fermentation

The addition of starter cultures during the production of fermented meat products results in the formation of certain aroma compounds as a result of the microbial fermentation. Lactic acid bacteria metabolize carbohydrates, resulting in a pH drop due to formation of lactic acid, which makes the taste of fermented meat products rather acidic. Fermentation of carbohydrates as well as lipolysis and proteolysis generate precursors for volatile odor compounds [18,71]. In addition, chemical reactions in the form of lipid oxidation along with the Maillard reaction and the Strecker degradation contribute to the odor and flavor of fermented meat products. However, the chemical reactions are limited due to processing parameters such as low temperature, low pH, etc. [71]. The important aroma compounds in fermented sausages were reviewed by [71], where it was reported that aldehydes are one of the most important chemical groups with hexanal being the most significant aldehyde, followed by pentanal, octanal, and 2-nonenal (grass, green, herbal odors [17]). The most important ketone is 2,3-butandione (buttery odor [17]), while carboxylic acids include acetic acid, butanoic acid, and 3-methylbutanoic acid (cheesy notes). Esters in fermented sausages include ethyl butanoate, ethyl 2-methyl butanoate, and ethyl pentanoate (fruity odor and caramel notes). The most important nitrogen compound is reportedly 2-acetyl-pyrroline (nutty, roasted), while low odor threshold-sulfur compounds include diallyl sulfide, diallyl disulfide, dimethyl disulfide, methional, and methionol (garlic, onion, cooked potato, cooked meat). Terpenes (vegetal notes) in fermented sausages originate from the use of spices, while phenolic compounds are formed during the smoking stage [71].

Interestingly, some volatile aroma compounds may have more than one mode of formation. For example, 2-methyl-3-furanthiol is not only produced during the heat degradation of thiamine, but also as a result of the Maillard reaction [31] and microbial metabolism, and has, thus, been detected in fermented meat products [72]. The high free amino acid content, low water activity, low pH, and long drying times are suggested to be responsible for the formation of 2-methyl-3-furanthiol in fermented meat products [31,73].

#### 2.3.4. Irradiation

The irradiation of fresh meat as a way of controlling spoilage and eliminating foodborne pathogens is approved in the United States by the Food and Drug Administration and the United States Department of Agriculture (mainly used on beef, pork, and poultry) [74], and in the EU by certain member states (mainly used on poultry, offal, frog legs, and animal by-products) [75,76]. The method is quite efficient. However, irradiated odors described along the lines of “pungent”, “rancid”, “rotten egg”, “sulfur”, “bloody”, etc., may develop due to lipid oxidation and degradation of sulfur-containing amino acids [3,56]. The most important volatile compounds formed include the hydrocarbons 1-heptene and 1-nonene (influenced mainly by irradiation dose), the aldehydes propanal, pentanal, and hexanal (influenced mainly by packaging atmosphere, i.e., with or without oxygen), and various sulfur-containing compounds such as dimethyltrisulfide and bismethylthiomethane (from the degradation of sulfur-containing amino acids) [56]. In order to prevent the development of off-odors due to irradiation, the use of low temperature, vacuum or inert gas packaging, and the addition of antioxidants are viable options [56].

#### 2.3.5. Cooking

The course of the Maillard reaction depends on the nature of the food product in question, i.e., moisture content, pH, reactants involved, and cooking temperature. The rate of the Maillard reaction increases with increasing temperature and decreasing moisture levels, meaning that the formation of the Maillard reaction compounds primarily takes place in the areas of the food that have been dehydrated by the cooking process [77].

According to [21], the cooking method is undoubtedly the most important extrinsic factor influencing the generation of volatile aroma compounds in meat. It has been shown that roasting enhances the formation of sulfur and carbonyl compounds, while stewing produces an entirely different aroma profile with odor compounds belonging to a variety of classes [3]. The cooking method additionally affects the formation of WOF. Microwave heating produces a higher degree of WOF volatiles than pan frying, grilling, or boiling. This has been attributed to the fact that grilling and pan-frying produce Maillard reaction products on the cooked surface, masking the WOF, or perhaps a retardation of the production of the volatiles responsible for WOF [78] (Section 2.1.2).

In addition to cooking method, cooking temperature also significantly influences the generation of cooked meat odor volatiles [3]. For example, it has been shown that pan-frying pork at 150 °C leads to domination of lipid-derived volatile compounds, whereas pan-frying at 250 °C leads to formation of predominantly Maillard reaction-derived volatiles [79].

The type of fat used for cooking, not surprisingly, influences the generation of cooked meat aroma. Frying meat in an oil with a high content of polyunsaturated fatty acids such as sunflower oil produces a high content of aliphatic aldehydes and more heterocyclic Maillard reaction compounds than cooking in olive oil, butter, or lard [80]. When frying in olive oil, which contains a higher proportion of monounsaturated fatty acids than sunflower oil, the volatile profile is dominated by lipid-derived volatiles. Frying in butter results in a high amount of high-carbon ketones, while frying in lard results in certain Strecker aldehydes and dimethyl sulfide found only in these samples [80].

The cooked meat aroma and flavor are further influenced by added spices [18] and smoking [3], the latter contributing compounds such as lactones and numerous different phenols to the volatile profiles of meat products [81].

### 2.4. Key Odor Compounds in the Meat of Various Species

Key odor compounds in cooked meat of different species were recently reviewed by [12] for beef, pork, chicken, and lamb. An overview is shown in Table 2. Generalized odor descriptors for compounds from the various chemical classes can be found in [17] and are listed in Table 1.

The significance of a volatile compound to the odor of a food is generally determined according to the OAV, which, as previously mentioned, is the ratio of the concentration of the volatile compound in the food divided by the odor threshold of the compound in an appropriate matrix. However, these odor thresholds and OAVs are determined for the individual compound, and it is recognized that the interaction between different volatile compounds results in a loss of aroma properties of the individual compound [17]. An additional issue is that the odor threshold is often determined with water as the matrix when the threshold in air might be more relevant [114].

It is well known that volatiles originating from lipid oxidation such as ketones and alcohols have high odor thresholds (mg/L range), aldehydes µg/L to mg/L range, while N- and S-heterocyclic compounds originating from the Maillard reaction and Strecker degradation have odor thresholds in the µg/L range [11]. The odor thresholds for the most important odor-active compounds in cooked meats were reviewed recently [103].

## 3. Methods for the Determination of Volatile and Odor Compounds

### 3.1. Overview

There are many methods available for analyzing the VOCs in meat. Most of them require the analysis of the sample via gas chromatography-mass spectrometry (GC-MS), because this method has many advantages over other methods. In combination with specific sample preparation techniques, it can be used for gaseous, liquid, and solid sample analysis. GC-MS is also highly sensitive and specific [115].

The following description of methods for the analysis of VOCs will be focused on meat and meat products from pigs and cattle, although most of the methods seem to be suitable for other kinds of meat as well. Moreover, only methods that are relevant in practice are included. This is decided based on the number of publications that appear for the corresponding search terms in PubMed and Google Scholar. Methods that are theoretically suitable but are scarcely used for the analysis of meat VOCs are not included.

### 3.2. Sample Preparation Techniques

The process of sample preparation is the most important step prior to the analysis of the volatile composition of the sample by GC [116]. Meat, being a heterogeneous sample matrix with many different components such as fat, muscle fibers, and connective tissue, usually needs to be minced and homogenized to be useable for analysis [11,117].

Although a variety of sample preparation techniques for the analysis of VOCs in meat are available (as reviewed by [11,117]), this review focuses on three methods that appear to be the most commonly employed, based on the number of publications listed in PubMed and Google Scholar.

#### 3.2.1. Solid-Phase Microextraction (SPME)

Nowadays, the SPME technique is possibly one of the most widely used sample extraction techniques for meat analysis and food research in general. It is a sorptive extraction technique. During the exposure of a thin fused silica fiber coated with a sorptive liquid polymeric material to the sample matrix, the VOCs contained in this matrix are adsorbed or absorbed in the fiber coating material and then are thermally desorbed in the inlet of a gas chromatograph. The sample is both extracted and pre-concentrated during this process [116].

There are two possible methods available for the exposure of the fiber to the sample. It can be carried out either by direct immersion into a liquid sample matrix at the bottom of the headspace (HS) vial (DI-SPME) or as a headspace extraction technique by adsorbing or absorbing the volatiles in the headspace over the sample in the vial (HS-SPME) [116]. Since DI-SPME significantly shortens the lifetime of the fibers, and the sample needs to be in the liquid phase, HS-SPME is the method of choice for the extraction of the VOCs in food, and consequently, in meat samples, as it has many advantages over other extraction methods [11].

The sample matrix can be in the gaseous, liquid, or solid phase when HS-SPME is used for the extraction of the VOCs. After placing the sample in a HS vial and sealing it with a pierceable septum, it is usually heated and agitated under controlled conditions. During this process, the VOCs in the sample reach an equilibrium between the sample matrix and the headspace of the vial. In the next step, the fiber is exposed to the VOCs in the headspace of the vial [116]. The VOCs diffuse from the headspace of the vial into the coating material on the fiber surface following the first partition coefficient. When the extraction process is finished, an equilibrium between the VOCs in the headspace of the vial and the coating material is reached. After removal of the fiber from the vial, it is thermally desorbed in a heated inlet of a gas chromatograph [116].

Advantages of HS-SPME include that the method can be completely automated and is relatively easy to use and reproduce in comparison with other meat VOC extraction techniques. Another advantage is that HS-SPME requires only a relatively small sample size to extract a high number of volatile and semi-volatile substances [11]. HS-SPME also has a high sensitivity and can reliably detect potential VOCs at concentrations in the range of ng/L [118].

A limitation of SPME in general is that the binding of the VOCs to the sorptive phase is strongly dependent on certain chemical properties (primarily the polarity and molecular weight) of both the VOCs and the sorptive coating material. However, there are many different fiber coatings available, covering a broad range of different molecular masses and polarities of the VOCs. Some fiber coatings are available in various thicknesses, broadening the detection range of possible VOCs even more [116].

Mostly two different fiber coating materials are used in meat volatile compound analysis involving HS-SPME: Carboxen/Polydimethylsiloxane (CAR/PDMS) with a coating thickness of either 85 µm or 75 µm and Divinylbenzene/Carboxen/Polydimethylsiloxane (DVB/CAR/PDMS) with coating thicknesses of 30/50 µm [11]. Both fibers are bipolar fibers, covering a broad range of possible VOCs with various chemical properties, especially the triple phase fiber DVB/CAR/PDMS. The CAR/PDMS fiber is recommended for low molecular weight compounds and trace-level volatile analysis, while the DVB/CAR/PDMS fiber is to be chosen for the analysis of an expanded range of VOCs (C3-C20) and for the analysis of volatile and semi-volatile flavor compounds [116].

However, HS-SPME still has limitations for molecules with high molecular masses, which cannot be analyzed with this technique. Furthermore, polar, very volatile, or thermolabile substances are reported to have a low extraction efficiency in general [11]. Another problem is that there are no fiber coatings available, which are polar enough for the extraction of very polar or ionic substance species. Hence, ionic, polar, and non-volatile substances need to be derivatized or otherwise altered before they can be extracted with the SPME technique [116]. Because of different extraction efficiencies of various VOCs, it is possible that volatile compounds with a very high extraction efficiency in the sample saturate the relatively low sorptive capacity of the fiber coating material and displace VOCs with a lower extraction efficiency, consequently leading to false results of the calculated concentrations. Another disadvantage of the SPME method is its susceptibility to mechanical destruction of the thin fiber; at the same time, new fibers are relatively expensive [11].

To circumvent some of the above-mentioned problems, there are new modifications of the SPME technique available, which aim to enhance the lifespan and the extraction efficiency of the fibers, as well as the overall sensitivity of the method. Examples of these new modifications are: SPME arrow and thin film SPME as well as the use of nanomaterials as fiber coating layers [11,116].

HS-SPME has been used successfully in the detection of the possible aroma compounds of both beef and pork and meat products such as sausages. Examples of analyzed beef and pork products with the HS-SPME technique are cooked Hanwoo beef [101], raw beef [119], ground beef [120], beef extract powder [121], cooked pork [122], minced pork [123], pork bellies [124], stewed pork [125,126], dry-cured ham [127], fried pork loin chops [80], and smoke-cured bacon [128]. For additional information, refer to Table 3.

HS-SPME can be used in the analysis of the volatile compounds of poultry as well, for example, turkey [129,130].

Another possible application of the SPME extraction method in the context of pork analysis is the detection of potential off-odors such as boar taint, which is mainly caused by the substances androstenone and skatole [131,132]. It might be possible to analyze pork and beef products for potential irradiation markers with the SPME technique as well [120,121,133]. In dry-cured Iberian ham, another off-odor, bone taint, is described and can also be analyzed with the SPME technique [99].

#### 3.2.2. Stir Bar Sorptive Extraction (SBSE)

The SBSE technique follows the same principle as the SPME extraction technique and can be seen as an extension of it. SBSE is used for the extraction and enrichment of VOCs from liquid sample matrices. In contrast to the SPME technique, which uses a fused silica fiber coated with a sorptive material, a glass-covered magnetic stir bar coated with PDMS is used in the SBSE technique [116]. Just like in the SPME technique, the VOCs are adsorbed into the PDMS coating during the incubation phase. The main advantage of SBSE is the capacity of the sorptive phase, which is enlarged significantly in comparison with SPME [116,134]. Whereas the extraction phase used for the SPME technique has an extraction volume of 15 µL, the one used in SBSE has an extraction volume of up to 125 µL. The result of this increased volume is a better phase ratio with an increased recovery of the VOCs, which leads to up to 250-fold lowered limits of detection in comparison to SPME [116,135].

One disadvantage of the SBSE technique is the lack of various coating phases for VOCs with different chemical properties. Up until now, only PDMS is commercially available [134]. Another problem is the need for a liquid extraction milieu, which is difficult to achieve with meat samples [134]. Nevertheless, one study [136] used the post-cooking exudate of cooked, cured pork ham samples for the extraction of the volatile compounds with the SBSE technique, which might be a suitable way to analyze cooked meat. Another study [137] used the SBSE technique to determine the compounds responsible for saffron aroma in flavored cured ham.

#### 3.2.3. Dynamic Headspace Extraction Techniques (DHS)

DHS is another common group of methods for the analysis of volatile compounds in meat samples. The VOCs contained in a solid or liquid sample matrix are vaporized by heating in a controlled flow of an inert gas. The performance of the extraction is dependent on the concentration and volatility of the VOCs contained in the sample. Other factors influencing the extraction efficiency are the sample size, the type of sorbent, the extraction time, and the flow rate of the carrier gas [11].

One advantage of DHS methods is their usefulness for the analysis of sample matrices containing volatile compounds at ultra-trace levels, because the capacity of the sorbent phase is relatively high in this group of methods. For example, volatile halogenated hydrocarbons in drinking water can be detected in concentrations in the range of ng/L [116]. In comparison to other extraction techniques, the process of sample preparation in DHS is relatively simple and solventless. DHS can be completely automated, and a wide range of sorbents are available [11].

A major disadvantage of DHS methods is their tendency to collect moisture, which could damage the whole GC-MS apparatus. It is possible to manage the moisture problems via dry purging, but it seems to be quite difficult in samples containing significant levels of moisture [11].

One DHS technique of particular importance for the analysis of VOCs from meat is called purge and trap (P&T). Prior to the general availability of sorptive extraction techniques, P&T was one of the most widely used methods for the extraction and concentration of volatiles and, in some use cases, it still is, since it can be applied for nearly every kind of sample matrix [11]. In P&T, the sample is initially placed in a trap-like device that contains sorptive materials adsorbing or absorbing and retaining the VOCs [117]. The next step is to heat the trap to desorb the volatile compounds from the sorbents into an inert gas stream and then transfer and inject them into the inlet of a GC column [11]. This technique has been used to analyze the VOCs in animal by-products [138,139,140], raw and cooked turkey [141], dry-cured pork shoulder [142], and raw beef [143]. The advantages of this technique are the high sensitivity for volatile compounds and the possibility of automation. The main disadvantage is moisture collection of the instrument, just like in other DHS methods [116].

### 3.3. Gas Chromatography (GC)

To analyze and separate volatile and semi-volatile flavor-contributing substances in meat, gas chromatography (GC) is widely used [91,92,101,136]. Additionally, the separation and analysis of non-volatile components in foods via derivatization is possible [144].

After the isolation of the VOCs from the matrix during the sample preparation, the VOCs are injected into the column of a GC apparatus. In principle, a GC device consists of an inlet, a column, and an outlet to which a detector is connected. The volatile compounds enter a stream of inert carrier gas after their evaporation in the inlet. Carried by this gas stream, they enter the column, which is coated with a coating material, acting as a stationary phase. Due to their interactions with the stationary phase, the VOCs are separated primarily based on their polarity and mass [116].

In modern laboratories, fused silica capillary columns are used, which require a well-controlled sample inlet system in order to provide the best possible results and to avoid overloading the column [116]. For the analysis of the volatile composition of meat, a splitless injection mode seems to be used in most published research articles [123,145,146]. This corresponds to recommendations for the analysis of trace-level compounds [116]. Another important consideration is the selection of the right column. It is particularly important to choose the correct polarity of the column. If the compounds are mostly hydrocarbons with many single bonds, a non-polar column should be used. If many double and triple bonds, as well as other atoms such as oxygen, sulfur, phosphorus, and nitrogen are contained in the molecules to be analyzed, the use of a polar column is preferable. In general, the polarity of the compounds should match as closely as possible the polarity of the stationary phase of the column. The use of a column with a 5%-diphenyl-95%-dimethyl-polysiloxane stationary phase is recommended for flavor compound analysis [116]. The use of 6%-cyanoprophylphenyl-94%-dimethyl-polysiloxane as inner coating material of the column is common in the analysis of meat VOCs as well [73,147]. This material is of intermediate polarity and is recommended for the analysis of VOCs [116]. Interestingly, columns with polyethyleneglycol as the stationary phase are used as well in some published articles [96,148]. However, this material is not particularly recommended for VOC analysis [116] and has a potentially lower resolution for this application in comparison to a column with a 5%-diphenyl-95%-dimethyl-polysiloxane stationary phase [101].

If two compounds have similar or nearly identical retention times and co-elute, it might be advisable to change to a column with a significantly different polarity and compare the results from the two different columns [149]. Other parameters such as the length, the inner diameter of the column, and the film thickness are important as well [116], but a more detailed explanation would go beyond the scope of this article.

Another consideration for the operation of a GC device is the selection of the carrier gas. Helium is used in most cases, although a shortage of natural helium is expected in the future and prices tend to increase. Switching to hydrogen as the carrier gas in GC systems might be a solution to this problem [116].

### 3.4. Mass Spectrometry (MS)

To identify and quantify the separated compounds in a GC-analyzed sample, a detector is needed. Although a variety of ionization detectors are available, for the detection of meat VOCs, MS seems to be the method of choice [11].

The broad use of 70 eV as the electron ionization energy allows the comparison of the resulting compound mass spectra with those in many commercial mass libraries for the identification of VOCs [150]. Commercial mass libraries often used for the identification of the VOCs in meat are the NIST library [92,125,136] and the Wiley library [96,124,149], although other alternatives exist as well, such as MassLab [151].

Another method for compound identification is the calculation of linear retention indices (LRIs) from the retention times of the corresponding peaks in the chromatograms. This requires the analysis of a standard series of homologous n-alkanes under the same analytical conditions as the sample [150]. The calculated LRIs are then compared with the results of previously published articles using similar analytical conditions. However, without a specific suspected substance corresponding to the peak in the chromatogram, no conclusions can be drawn regarding the type of substance identified. Thus, the calculation of the LRIs is an additional aid for the identification of the substances corresponding to the peaks based on their mass spectra. Useful internet sources for published LRIs of VOCs are webbook.nist.gov (accessed on 30 August 2022), pherobase.com (accessed on 30 August 2022), and flavornet.org (accessed on 30 August 2022).

The identification process of the compounds can also be carried out by comparison of their retention times and mass spectra with the ones from certified commercially available reference compounds analyzed under the same analytical conditions [101,136]. For reliable identification, the retention times and mass spectra of the compounds should match those of the reference substances as closely as possible. For economic reasons, this comparison with authentic standards seems to be useful only if concrete assumptions about the types of substances are available, such as a high match factor of the mass spectra with those from the libraries.

Quantification is carried out by analyzing internal or external standards and calculations of the different response factors for each compound based on their peak area [117].

### 3.5. Gas Chromatography-Olfactometry (GC-O)

For the detection of odor-active compounds in meat and foods in general, the use of the human nose as a detector seems to be suitable in addition to the analysis and detection of VOCs by GC-MS. This can be carried out via the GC-O technique. Moreover, in order to identify the aroma characteristics and the intensity of the detected VOCs, an accompanying investigation by GC-O is mandatory [152]. For trained assessors, it is possible to provide a description of each perceived odor and its relative intensity in parallel to the detection of the substances by GC-MS with this method [99].

In principle, a sniffing device is coupled to a GC system with a split at the end of the chromatographic column. Trained assessors sniff the eluate, which is split in a specific ratio between both the sniffing port and the MS detector, in parallel with MS detection. At the sniffing port, the eluate from the column is combined with a heated and additionally humidified inert gas [116].

There are two main techniques of analysis used in GC-O: successive dilution of an aroma extract until no odor is perceived at the sniffing port, and the recording of the intensity of the aromatic compounds of an aromatic sample as a function of time [116]. The latter group of methods is termed time-intensity methods [152]. It is possible to create an aromagram complementary to the chromatogram by documenting the peak-to-odor correlation. This provides the ability to derive an OAV specific to each substance [116].

GC-O was successfully used to determine odor active compounds in pork [91,92,125,126], beef [82,83], chicken [104,105,106], and lamb [107,108]. Table 2 provides further information on compounds identified with this technique.

### 3.6. Electronic Noses

The analysis of odor and flavor in food, traditionally through organoleptic testing, is very time and cost intensive. Different electronic systems such as electronic noses or electronic tongues could be a cheaper alternative for this [153]. An electronic nose simulates the physiological processes in the human olfactory system. The main idea behind this technique is to transmit the chemical reaction of receptor-binding of an odor-active substance, as it would occur in the human nose, to a digital electronic signal. Just like in the human olfactory system, different specific sensors for different chemical compounds such as amines, aldehydes, alcohols, esters, and ketones are needed [154]. The first mechanical device used for flavor classification was described in 1961 [155]. The first electronic model olfactory system was presented in 1982 [156]. In the 1990s, the electric nose began to be used for meat testing [157,158]. Compared to other methods, the biggest advantage of the electronic nose is that it is a non-invasive technique.

There are two main components of a working electronic nose: the gas sensor, which acquires the data, and the neural network for data analysis in the background [158]. In early applications, huge and error-prone classical chemical sensors were used [159]. The next generation of gas sensors were metal oxides [153,160]. The newest generation of sensors are organic field-effect transistors (OFETs) [161]. Another sensor type, which also works at room temperature, is the polymer sensor [162]. Now, the focus is placed on the development of small devices and a real-time monitoring of odors [159].

There are several studies about different purposes for measurements with electronic noses. In most studies, the purpose is to assess the freshness of the meat product. In this case, freshness is defined as the absence of different signs of spoilage such as lipid oxidation, enzymatic autolytic processes, microbiological spoilage, and an undesirable flavor profile [163,164,165,166,167].

Other objectives include the detection of defects in the meat during the production chain before slaughtering, such as boar taint [168]. More examples for the use of this technique in the analysis of meat are to identify the components used for feeding the animal [169] and to detect errors in the postmortem treatment [170]. Moreover, electronic noses can be used to determine the ripening time of meat [169] and the length of the storage period [167]. There is a considerable impact of sample storage conditions on the results. Frozen storage at −21 °C changes many parameters, so this condition is possibly not ideal for testing with electronic noses [167].

A differentiation of species and breeds [171,172,173,174] and adulteration with meat from other species [173] is also detectable by electronic noses.

Meat from different animal species was examined in various studies. An overview is shown in Table 4.

Even though electronic noses have been known for 30 years with a constant improvement in the quality and functionality of sensors and a decrease in costs, it is crucial to point out that electronic noses have not yet been able to replace human sensory panels completely. The major reasons for this are the need of a high number of various sensors, the complicated way of sampling, and the large amount of data to be dealt with. New sensor technologies, powerful systems for data analysis, and a self-learning artificial intelligence-based system for data analysis will open a wide, new field for using electronic noses. However, well-trained human sensory panels will still be necessary for the calibration of future electric noses.

### 3.7. Sensory Analysis of Meat

Sensory examination of food is an important tool for the determination of product quality and consumer acceptance. This applies to meat as well. As previously discussed, differences in cooking techniques greatly influence the perceived characteristics of the meat. As mentioned earlier, Maillard reactions are related to the surface temperature of meat during cooking and greatly affect odor, flavor, and color. Sensory examination can be used for the evaluation of these sensorially perceptible properties of meat and meat products [183].

There are three key eating quality properties for meat: tenderness, flavor, and juiciness. In addition, the color of meat is an important value for consumer satisfaction [184]. Whereas parameters such as color, tenderness, and water holding capacity are measurable by different instrumental methods, human sensory perception is essential in order to determine flavor and odor. Sensory panels are considered an established possibility for quality control in the food processing chain. Up until now, it has not been possible to replace the use of a trained sensory panel with artificial olfactory systems such as an electronic nose (see Section 3.6).

To obtain reproducible and statistically significant results, correct sampling and sample storage are important factors. Samples should be taken from representative animals and positions of the carcass. Packaging material should be inert and with no inherent smell and taste such as trays and foil made from PA (polyamide), PP (polypropylene), or PET (polyethylenterephthalate). For long term storage, frozen conditions are necessary [185,186].

A number of different techniques to assess sensory profiles of food taste and odor exist. These can be divided into two groups: the analytical tests and the affective tests. Since affective tests focus on detecting subjective preferences of consumers, they will not be discussed further, although they play an important role, especially during the process of the development of new food products [187].

The analytical tests can be subdivided into the discrimination tests and the descriptive tests. Discrimination tests are basic analytical sensory tests. They are designed to check if the panelists can detect any differences between two samples. Descriptive tests, which ideally require a panel of trained sensory panelists, are performed to determine and quantify the organoleptic properties of the food products. Descriptive tests seem to be the most capable tools for the examination of the sensory characteristics of food products because they yield the greatest amount of information, and the interpretation of the results is intuitive [187].

Quantitative Descriptive Analysis (QDA) is probably the most widely used and most consolidated descriptive technique of sensory examination [183]. With this method, sensory characteristics such as color, odor, taste, and texture of a food can be evaluated both qualitatively and quantitatively. One study [188] used a panel consisting of ten trained and experienced panelists to examine grilled meat and meat products from entire male pigs for the presence of boar taint. A different study [189] used QDA to compare flavor, tenderness, and juiciness of broiled loin chops from two different cattle breeds. Flavor and odor of chicken meat [185,190] as well as lamb [183] have been examined with this method as well.

Another category of sensory tests is called dynamic sensory methods. These methods consider the perception of the organoleptic properties of food changes over time of consumption. Important examples for meat are the time-intensity (TI) and the temporal dominance of sensation (TDS) methods. Up until today, these methods have seen limited use in the determination of the flavor of meat [191]. Nonetheless, the TI technique was used by a group of authors to evaluate the sensory attributes of dry-cured ham [192,193] and dry-cured loins [194]. This method was also used to examine the role of salt and the content of fat on the temporal perception of texture and flavor in cooked bologna sausages [195]. The effect of salt replacers on the texture and flavor of grilled sausages has been investigated with TDS [196]. To compare TDS with TI, both techniques were used simultaneously in two of the previously mentioned studies [193,194]. Both the TI and TDS techniques should be used if a profound sensory examination of a given sample is the objective of the evaluation, because the results of the two methods are complementary [191].

Finally, it is important to note that the relationship between individual laboratory-based measures and sensorial properties is not 1:1. By design, conventional laboratory-based methods provide objective and surrogate data for a consumer’s subjective response to the sensorial properties of meat [184].

## 4. Conclusions and Perspectives

Cooked meat volatile and odor compounds are made up of a wide variety of VOCs, e.g., hydrocarbons, alcohols, aldehydes, ketones, carboxylic acids, esters, including lactones, ethers, furans, pyridines, pyrazines, pyrroles, oxazoles and oxazolines, thiazoles and thiazolines, thiophenes, and other sulfur- and halogen-containing compounds. Upon cooking, reactions leading to the formation of meat aroma include lipid oxidation, the Maillard reaction, Strecker degradation, thiamine degradation, and carbohydrate degradation, as well as the interactions between reaction products. Meat volatile composition is determined by both pre-slaughter and post-mortem factors such as species, breed, sex, age, feed, muscle type, chiller aging, and type of cooking or processing method (e.g., curing and fermentation).

Pre-slaughter, feeding, importantly, has the ability to influence the odor of cooked meat by altering the fatty acid composition of the meat, particularly meat from monogastric animals. Feed composition also makes it possible to introduce desirable plant-derived compounds into the meat. Post-mortem handling of the meat at any stage of the process from slaughter to the table will influence the aroma of the cooked meat or finished meat products. It might be of significance to bring further awareness to consumers as well as to the catering industry regarding how the type of fat used for cooking will influence aroma generation, and how cooking procedures such as grilling and pan-frying can be used to mask or delay development of WOF, and thus, reduce food waste.

When evaluating meat odor, both the qualitative properties as well as the quantities of the VOCs must be considered. Likewise, the composition of the food matrix is also significant when deciding on an appropriate method for analysis. The sample preparation methods most commonly used for the analysis of meat VOCs include SPME, SBSE, and DHS, while the most important analytical techniques are GC-MS, optimally in combination with GC-O, as well as electronic noses. Both the sample preparation methods and the analytical techniques will continue to evolve in the future and to play an important role for the detection of meat VOCs. For example, new modifications of the SPME technique have recently been developed to improve its capabilities as well as reduce its drawbacks. GC-MS technology is also under continuous development, and new modifications are applied constantly. The future potential for the electronic nose is also promising. Within the next few years, if the transfer from use under standardized laboratory conditions is successful, a cheap, accurate, and robust method will be available for routine food analysis in industry and food safety agencies. This will require developments in sensor material and technique, a database with reference material to increase specificity, and artificial intelligence algorithms for data analysis. With real-time measurements a few seconds before packaging, it would be possible to increase consumer satisfaction and safety of meat and meat products. However, though analytical methods are presumed to continuously improve in the future, trained sensory panels will expectedly remain a necessity for the optimal evaluation of the odor and flavor of food, including meat and meat products.

## Figures and Tables

**Table 2:** Types and relative contents of characteristic odor compounds in different thermally processed meats detected by gas chromatography-olfactometry [12] *.

	Classification	Volatile Compound	Relative Content (µg/g)	Odor Descriptors
**Beef**	Aldehydes	Hexadecanal	81.41	Cardboard [82]
[82,83]		Nonanal	5.39	Fat, citrus [82,83]
		Hexanal	2.08	Grass, fat [82,83]
		Benzaldehyde	0.12	Almond, burnt sugar [82]
	Alcohols	Z-9-octadecen-1-ol	0.34	Fatty, animal ^1^
		1-octen-3-ol	0.16	Mushroom [82,83]
	Ketones	3-Hydroxy-2-butanone	0.70	Buttery, creamy, fatty, sweet [84]
		2-Octadecanone	0.55	Green ^1^
	Carboxylic acids	Hexanoic acid	0.89	Sweat [82]
		2,4-Hexadienoic acid	0.21	Acrid [85]
		Nonanoic acid	0.03	Fatty, cheese [86]
	Esters	Ethyl acetate	50.58	Pineapple [82]
		Ethyl 9-hexadecenoate	0.18	Fruity [87]
	Furans	5-Methyl-2-acetylfuran	0.71	Nutty [88]
		Tetrahydrofuran	0.66	Butter, caramel [89]
	Heterocyclic	3,5-Diethyl-1,2,4-trithiocyclopentane	2.85	Beef aroma [90]
**Pork** [91,92]	Aldehydes	Nonanal	2.86	Fatty, floral, wax [93]
		Benzaldehyde	2.53	Bitter almond [93]
		Octanal	1.97	Fatty, pungent [93]
		Trans-2-nonenal	1.47	Cucumber, farinaceous, greasy, grassy [94,95]
		Heptanal	1.25	Fatty, putty [93]
		Hexanal	0.95	Green, grass [93]
	Alcohols	3-Methyl-1-butanol	3.10	Pungent [96]
		Hexanol	1.11	Woody, cut grass, chemical-winey, fatty, fruity, weak metallic [54]
		1-Octen-3-ol	0.83	Mushroom [93]
		3-Methyl-3-buten-1-ol	0.34	Sweet fruity ^1^
	Ketones	2-Butanone	0.83	Chemical, burnt, gas, chocolate [97]
		2-Heptanone	0.80	Citrus, grapefruit, floral, fruity, spicy, cinnamon [54,98]
	Esters	γ-Butyrolactone	0.96	Creamy, pleasant, sweet [84]
		Ethyl 2-methylbutanoate	0.35	Fruity, strawberry-like [99]
	Carboxylic acids	Hexanoic acid	0.81	Goaty [11]
		Nonanoic acid	0.25	Fatty, cheese [86]
	Sulfur compounds	Methional	1.74	Cooked potato, roasted [100]
		Dimethyl disulfide	1.24	Moldy, pungent, rubbery, onion-like [101]
	Pyrazines	2,5-Dimethyl pyrazine	0.24	Nutty, musty, earthy, roasted, cocoa powder [102]
	Furans	2-Pentylfuran	1.29	Green bean, butter [54]
**Chicken**	Aldehydes	P-methoxybenzaldehyde	20.90	Anisic ^1^, hawthorn-like [103]
[104,105,106]		Benzaldehyde	9.88	Almond, bitter almond, burnt sugar [82,93]
		Nonanal	0.73	Fatty, citrus, floral, wax [82,83,93]
	Alcohols	1-Octen-3-ol	0.06	Shiitake mushroom [106]
	Ketones	P-methoxypropiophenone	0.39	Musty, anisic ^1^
	Esters	Trans vinyl cinnamate	0.92	NR ^2^
	Furans	2-Pentylfuran	0.81	Green bean, butter [54]
		2-Acetylfuran	0.21	Butter, meaty [103]
**Lamb**	Aldehydes	Hexanal	109.23	Apple, leaf, delicate [107]
[107,108]		Heptanal	31.32	Nutty, fruity green [107]
		(E)-2-nonenal	30.09	Fatty, paper [103]
		Nonanal	18.25	Fatty, rancid [107]
		Benzaldehyde	13.09	Almond, bitter almond, burnt sugar [82,93]
	Alcohols	Hexanol	12.42	Woody, cut grass, chemical-winey, fatty, fruity, weak metallic [54]
	Carboxylic acids	4-Methylnonanoic acid	316.73	Sweet muttony or goaty [109]
		4-Ethyloctanoic acid	186.22	Sweet muttony or goaty [109]
		Acetic acid	5.09	Pungent, acidic, cheesy, vinegar [84]
	Esters	Ethyl dodecanoate	6.18	Fatty [110]
	Furans	2-Methyl-5-(methylthio)furan	36.09	Meat, onion [111]
		2-Pentylfuran	24.21	Green bean, butter [54]
	Pyrazines	2,3,5,6-Tetramethylpyrazine	15.52	Chocolate-like [112]
	Sulfur compounds	Benzyl methyl sulfide	4.88	Roasted, muttony, burning [113]

* Reprinted in adapted form from Food Research International, 151, Sun, Wu, Soladoye, Aluko, Bak, Fu, and Zhang. Maillard reaction of food-derived peptides as a potential route to generate meat flavor compounds: A review, 110823, 2022 with permission from Elsevier. ^1^ Odor descriptor(s) retrieved from thegoodscentscompany.com. ^2^ NR Not recorded.

**Table 3:** Conditions of the analysis of VOCs in meat via SPME applied in ten research articles on the subject.

Reference	Type of Meat Sample	Sample Preparation	Weight [g]	Vial	Temperature During Exposure [°C]	Duration of Sample Equilibration [min]	Duration of Fiber Exposure [min]	Compounds Identified (In Total/Odor Active) ^1^
[101]	Beef	Powdered	1	40 mL vial	60	10	30, 45, 60	96
[119]	Beef	Diced	10	20 mL vial	4	0	20	35
[120]	Beef	Grounded	2	10 mL vial	40	10	10	2 (targets)
[121]	Beef extract powder	Irradiated	5	20 mL vial	40	0	40	61
[122]	Pork	Trimmed and cut	30	100 mL reagent bottle	60	5	30	96
[123]	Pork	Minced	2	20 mL vial	40	15	30	41
[124]	Pork	Not specified	2	20 mL vial	65	0	60	30
[125]	Pork	Not specified	5	40 mL vial	60	20	40	139/29
[126]	Pork	Not specified	5	40 mL vial	60	20	40	60/26
[127]	Dry-cured pork ham	Homogenized	10	40 mL vial	50	30	60	>40/12

^1^ If gas chromatography-olfactometry was subsequently used, the number of odor-active compounds was given in the articles. The number of all identified VOCs was often only estimated in these cases.

**Table 4:** Overview of studies where meat volatiles and odor were detected by electronic noses.

Species	Aim	References
Pork	Flavor profile, quality	[153]
	Lipid oxidation	[165]
	Boar taint	[168]
	Feed composition and ripening time	[169]
	Flavor profile	[171]
	Adulteration	[173]
	Oxidation	[175]
Beef	Spoilage	[163]
	Lipid- and protein oxidation, flavor profile	[167]
	Effect of processing parameter on odor profile	[176]
	Flavor profile	[171]
	Adulteration	[173]
	Freshness evaluation	[177]
	Pathogen detection (reviewed)	[162]
Poultry	Lipid oxidation	[165]
	Freshness evaluation	[177]
	Cooking state	[178]
	Flavor profile	[179]
	Freshness evaluation	[180]
	Biogenic amine index	[181]
Other	Flavor profile (donkey)	[171]
	Flavor profile (sheep)	[171]
	Flavor profile (llama)	[172]
	Volatile composition (goat)	[170]
	Bacterial contamination (goat)	[182]

## Data Availability

Not applicable.
